# Data-Driven Packet Loss Estimation for Node Healthy Sensing in Decentralized Cluster

**DOI:** 10.3390/s18020320

**Published:** 2018-01-23

**Authors:** Hangyu Fan, Huandong Wang, Yong Li

**Affiliations:** Department of Electronic Engineering, Tsinghua University, Beijing 100084, China; fhy14@mails.tsinghua.edu.cn (H.F.); whd14@mails.tsinghua.edu.cn (H.W.)

**Keywords:** failure detection, distributed system, gossip protocol, stochastic packet loss

## Abstract

Decentralized clustering of modern information technology is widely adopted in various fields these years. One of the main reason is the features of high availability and the failure-tolerance which can prevent the entire system form broking down by a failure of a single point. Recently, toolkits such as Akka are used by the public commonly to easily build such kind of cluster. However, clusters of such kind that use Gossip as their membership managing protocol and use link failure detecting mechanism to detect link failures cannot deal with the scenario that a node stochastically drops packets and corrupts the member status of the cluster. In this paper, we formulate the problem to be evaluating the link quality and finding a max clique (NP-Complete) in the connectivity graph. We then proposed an algorithm that consists of two models driven by data from application layer to respectively solving these two problems. Through simulations with statistical data and a real-world product, we demonstrate that our algorithm has a good performance.

## 1. Introduction

Clustering technologies leverage a set of connected computers to work as a single system [1], which improves performance, fault-tolerance and scalability of the system. It is extremely important in areas such as sensor networking, clouding computing, centralized controlling, etc. Compared with centralized clustering technology, the decentralized cluster has many advantages such as no single point bottleneck, no single point of failure, more flexibility [2]. However, it faces many challenges, one of which is failure detection [3]. Failure detection technologies use mechanisms such as heartbeat and timeout to provide failure sensing and troubleshooting approaches for the clusters and further make them failure tolerable [4]. Different with centralized clusters, in decentralized clusters there is no fixed supervisor who is responsible for failure detection and troubleshooting, leading to a more complicated failure detecting problem.

Existing failure detection methods [5,6,7,8,9,10,11,12] are designed to detect completely unreachable nodes, e.g., died or disconnected. A commonly used failure detection approach in a cluster is that, monitors estimate the state of each node based on the ϕ FD each node in the cluster is monitored by a set of other nodes. The monitor nodes send heartbeat requests to the target node and expect for heartbeat responses to obtain the link state between monitor and its target. If ϕ FD is adopted, monitors assume that the interval of heartbeat responses obey normal distribution. Not receiving a heartbeat response within an expected interval, they start suspecting the target to be unreachable. Then, they use gossip protocol [13] to spread the unreachability event to the rest of nodes. However, there are two main drawbacks of this kind of implementation. First, the existing failure detectors [5,6,7,8,9,10,11,12] cannot correctly detect a partially working node, e.g., a node which randomly drops packets. In this case, different monitors may give out different detection results on this node. Second, because gossip is a weak consistency protocol, it cannot properly deal with conflicts on reachability state, which is possible to lead to the corruption of the system. Due to what we have tested, one certain malfunction breaks a cluster with a size of 10 into pieces easily.

In this paper, we analyze the causes of the problem of the current work. As the ϕ FD and other failure detection mechanisms proposed in [5,6,7,8,9,10,11,12] are not suitable for the scenario of stochastic packet loss, we propose an algorithm that can estimate the severity of packet loss of a link between two nodes based on the statistical information of TCP protocol, the round-trip-time (RTT). Further, we propose a model that can sense a node’s healthy status from anywhere of the cluster without the limitation of any node to be always reachable, choose a unique leader without election process and make more reliable decisions about removing faulty nodes.

The contribution of this paper can be summarized as follows.

- First, we proposed a model to formulate the problem. Instead of modeling a link state to be reachable or unreachable, we model the link state to be healthy or unhealthy considering about unstable link. With a graph representing nodes and link states, the faulty nodes are found by solving a max clique problem(MCP). Moreover, we discuss the transitivity of the link state. For scenarios where link states have transitivity, we simplified the NP-Complete MCP to a linear complexity problem; For other scenarios, we proposed a square complexity heuristic algorithm which can find a maximal clique.
- Second, we proposed a data driven algorithm that solve the reliability issue in this specific case. Our algorithm uses an evaluation model to evaluate the link state basing on data from application layer. Basing on the evaluation results, the decision model takes care of the leader uniqueness issue and infers the faulty nodes.
- Finally, extensive simulation results demonstrate that our approach is highly adaptable. Basing on statistical data, the F1 score of the link evaluation method reaches more than 90%. And our implementation makes a real-world product stably run for more than a week while some of the the packet loss failure is injected in some of the nodes.

The rest of the paper is structured as follows. In Section 2, we introduce the related work in failure detection and troubleshooting on decentralized clusters. In Section 3, we present the formal model of our problem. In Section 4, we introduce our PingBased algorithm for enhancing the availability of decentralized cluster. In Section 5, we extensively evaluate the performance of our proposed algorithms compared with existing algorithms.

## 2. Related Work

### 2.1. Akka Cluster

A widely-used and well-recognized solution to the problem of failure detection and troubleshooting in decentralized cluster is a framework named Akka [14]. In Akka, each node is monitored by a number of other nodes with the technology of the ϕ FD. Each node in the cluster holds a reachability table and uses gossip protocol to keep consistent with other nodes. If one monitor detects that some node is faulty, it announces this event to the rest of the nodes by updating its own reachability table and make it consistent with the rest of nodes using gossip protocol. If a member is marked as faulty and has been broadcasted to all the other nodes, these nodes will then determine whether themselves shall be responsible for troubleshooting with a non-electoral leader determination algorithm. If different monitors have different outcome on whether a node is faulty, a pessimistic algorithm is adopted that nodes are only treated to be healthy if all its monitors say it is healthy, which in other words if any one of its monitors announces its faulty it will be treated as unhealthy.

### 2.2. Other Link Failure Detecting Algorithms

Besides ϕ FD, there are other algorithms aiming at detecting link failures. Analogous to ϕ FD, Xiong et al. [9] and Liu et al. [11] assume that the interval of heartbeat responses follows exponential distribution or Weibull distribution and calculate the probability of time interval between the current time and the time of last heartbeat response. If the probability is lower than a threshold, the monitor suspects the monitored node. Tomsic et al. [10] uses two windows with different sizes to collect the intervals of heartbeat messages. By comparing the current time and a predicted next receipt time calculated based on these two windows, the monitor decides whether the remote node is reachable or not. Turchetti et al. [12] proposes an IFDS framework which can handle multiple concurrent applications with different QoS requirements, whose purpose is different from us.

### 2.3. Packet Loss Measurement

Packet loss measurement of TCP has been studied in a number of works [15,16,17]. Sommers et al. [15], Wu et al. [16] predict the packet loss rate with implementations in routers where they can acquire the low level sequence and acknowledge numbers of the TCP/IP stack which cannot be obtained by applications. Basso et al. [17] provide a application layer estimation on packet loss rate. However, it assumes the RTT is a constant. In addition, it mainly aims at end users who download stream from a remote server, which is different from our work.

### 2.4. Other Works About Fault-Tolerance

Besides the solution above, there are also other works that focus on fault-tolerance in related fields. For example, Sun et al. [18], Cerulli et al. [19], Yim et al. [20] mainly aim at failure-tolerance in a more specific area of sensor networking. R. Şinca et al. [21] focuses on digital systems and implements fault-tolerant mechanisms on the hardware field. However, targets of these works are different from our work.

## 3. System Overview and Problem Formulation

### 3.1. System Overview

**Figure 1:**
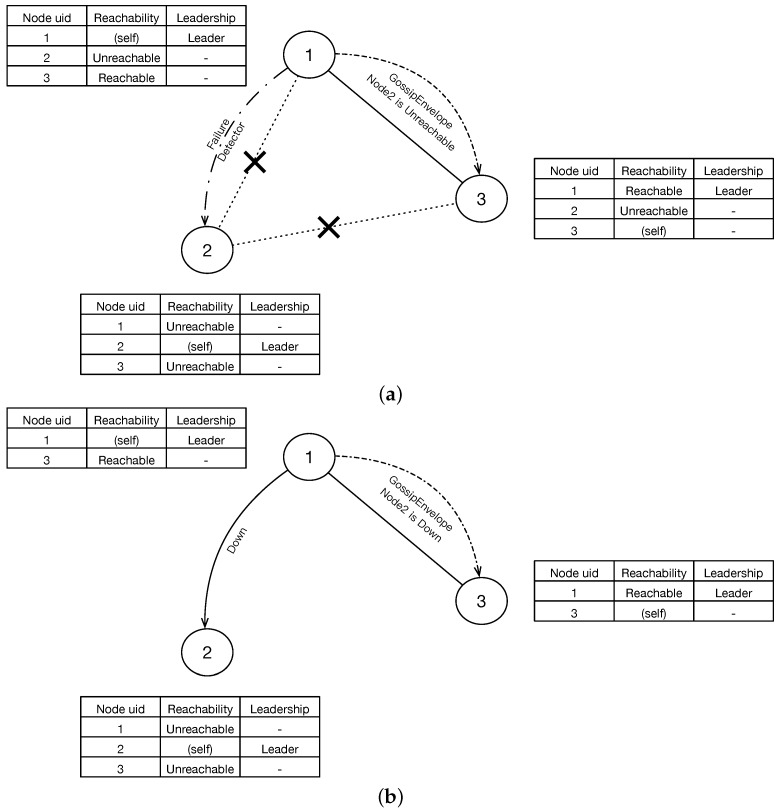
System overview of decentralized clusters that use gossip consensus protocol to manage members. (**a**) shows the member states, leadership and gossip process when detects a failure; (**b**) shows the down process of a leader.

In this section, we give an overview of our system of sensing healthy status of nodes in decentralized cluster based on Akka. Figure 1 shows the system overview of decentralized cluster services such as Akka cluster. Specifically, this system manages its members by maintaining a globally consistent member state table. To keep globally consistent, each node uses the Gossip Protocol [22] to repeatedly replicate its state to a randomly selected neighbor. To detect and handle node failure, each node in the cluster implements a heartbeat based failure detector to detect the reachability to some node in its neighbor. If one detects a failure, it will mark this node in its own member state table and try to gossip it to the rest of nodes in the cluster. Then a temporarily selected leader will handle this issue. We next make an expression of these processes in detail.

#### 3.1.1. Gossip Based Membership

In order to correctly cooperate with other nodes in the cluster, each member in the cluster holds a table that contains the states of all the members in the cluster and uses Gossip protocol to make this table globally consistent. The status of the member consists of two elements, i.e., working state and reachability state. These two elements stand for whether the node is working and whether the node can be reached, respectively. To make the state globally consistent, a node periodically exchanges its state to a random neighbor. If their exchanged states are different, the node with state of older version will update its state to the newer version. To add or remove a member, a node can simply modify its member table. After that, it gossips its member states to other nodes. When the member state meets the consistency, this add or remove action is finished.

#### 3.1.2. Failure Detection

Failure detection mechanism is used in the system to detect link failure, which further makes the cluster aware of node failures. To detect failure each node implements a failure detector. They use heartbeat or other mechanisms to keep monitoring a number of remote nodes selected by a specific principle. If a failure is detected, the monitor node will immediately mark that node by setting the reachability status of it to be Unreachable and use gossip protocol to ensure the entire cluster finally noticing this issue. Unlike the ϕ FD adopted by Akka, the failure detection mechanism in our work has an extra feature of estimating the severity of stochastic packet loss.

#### 3.1.3. Leadership

If an arbitrary node could decide whether a new node can join or leave, i.e., insert or remove node into or from the global membership table, it is possible to cause problems, e.g., difficulties in consistency of membership or logical issue in application. For example, if an arbitrary node could decide so, this node can do the leaving action by directly removing itself from the its own member table and gossip this table to the cluster. However, before the member table is globally consistent, some other members may not notice this leaving action and keep communicating to the leaving node. This may cause further issues like logical confusion. To avoid these problems, a temporary leader is selected to deal with actions affecting cluster’s size. For adding or removing a node to or from the cluster, the leader does this action gracefully with the following steps. First, instead of suddenly adding or removing a member from the cluster, the leader sets an intermediate working state to this member. Then the leader gossips its state to the cluster. After that, the leader waits until the member state is converged. Converged state means that the member state is consistent in the cluster so that all the members are conscious about the further action of this leaving or joining member. When the leader confirms the consistency of the intermediate state, it finally does the action of inserting or removing the member to or from the member table. The leader selection should be non-electoral to avoid being centralized. In Akka, the process is that a node considers a reachable node with the smallest unique identity to be de leader. The unique identity can be, for example, IP address and port. If the member states of all the members are consistent, it is expected that only one leader exists in the cluster at a time. And because the leader actions are only done when the leader obverses that the member state is converged, all the members will keep pace with the leader. In this way, the member state can always be easy to converge. Furthermore, the application can easily get the message of a member being joining or leaving. Thus, this approach is demonstrated to be a reliable way for changing cluster size.

#### 3.1.4. Downing

The leader has to deal with one more case. If a member is marked as unreachable, the leader will stop the leader action until it is recovered or forcibly removed. This is because if the leader has no access to an arbitrary node, it believes that the member state cannot be consistent (because the leader cannot replicate its state to this unreachable node). We can see that a member being marked as unreachable will block the leader action which has a critical function on the joining or leaving behaviors of members. Down mechanism is thereby put forward to eliminate the long-term blocking issue. With this mechanism, if a leader believes that the unreachable nodes are no longer available, it will forcibly remove the unreachable nodes from the cluster so that the cluster would work as normal.

### 3.2. Mathematical Model

Table 1 shows the model of our system. The cluster is represented by a set of *n* nodes denoted by *V*. The set of actual network state are denoted by $S_{n}$. The state of each node and each link are represented by $s_{n}$ and $s_{l}$, respectively. Each node manages a table of member states represented by the function fs. Nodes use Gossip protocol to make member states globally consistent. To select a leader, a node first selects a set of candidates basing on $r_{c}$, namely candidate rule. And it uses the function of h(v) and l(v) to determine a leader, where h(v) is used to get a unique identity of *v* and l(v) is used to return who is the leader. Leader removes a faulty node basing on the function of $r_{d}$, namely downing rule.

Particularly, we model the link in our system to be undirected [19,23], i.e., $s_{l}\left(v_{i},v_{j}\right)$ is equal to $s_{l}\left(v_{j},v_{i}\right)$. In addition, we only consider the scenario where the majority of the nodes in the cluster work,

(1) $$V_{h}:=\left\{v:v\in V,s_{n}\left(v\right)=Healthy\right\},\;\left|V_{h}\right|>\frac{\left|V\right|}{2}$$

We next talk about an important property of connectivity between nodes, i.e., transitivity. With the property of transitivity, no partial connectivity is appeared in the topology. This is to say two healthily connected end-nodes have the same link states to any other end-nodes. In traditional network this property is applicable because the endpoints do not have the ability to forward messages to others and the route protocol will eventually take care of the partially connecting issue. However, in case of topology like ad-hoc networking where endpoints are responsible for forwarding data or in case that unfair QoS is adopted, this property may not be applicable then. Therefore, we classify our algorithm into two cases divided by the applicability of this property. The transitivity can be described as the following formula:

(2) $$\exists v_{i},v_{j}\in V,v_{i}\neq v_{j},s_{l}\left(v_{i},v_{j}\right)=Healthy,\Rightarrow \forall v_{k}\;s_{l}\left(v_{i},v_{k}\right)=s_{l}\left(v_{j},v_{k}\right)$$

### 3.3. Problem Formulation

The main weakness of gossip based membership management is that once a node receives a newer version of member state from a valid sender it will merge the state into its own state with only a simple conflict avoidance logic. This influences little on a normally working cluster. However, in some cases, a problematic but valid node gossiping corrupt member state can bring severe problem to the cluster. Stochastic packet loss is a typical case of a node being problematic but valid. In this case, the problematic node with unstable links to other nodes may mark a part of other nodes as Unreachable uncertainly if the failure detection mechanism is unreliable. Moreover, unlike network partition issue, this node still has possibility to gossip its globally incorrect member state. If the incorrect member state corrupts the cluster, it will cause at least 2 fatal problems:

1. The leader may remove normal nodes that are marked as unreachable by the problematic one if the downing rule is not reliable.
2. There may be more than one nodes assume themselves to be leaders if all the normally working nodes with smaller unique id are marked by the problematic node.

More specifically, a faulty node $v_{f}$ marks a set $V_{x}\subseteq V\setminus \left\{v_{f}\right\}$ of nodes as Unreachable by the process of $\forall v\in V_{x}$ let $fs(v_{f},v)\leftarrow Unreachale$, and GossipTo $v_{j},v_{j}\in V\setminus V_{x}\setminus \left\{v_{f}\right\}$. Finally, the member state might converge to a result that $\forall v_{i},v_{j},v_{k}\in V\;s.t.\;fs\left(v_{i},v_{k}\right)=fs\left(v_{j},v_{k}\right)$. The commonly used original implantations of $r_{c}$ and $r_{d}$ are shown in Table 2. In this case, the uniqueness of leader cannot be guaranteed. As a result, it may happen that 2⩽k⩽n (recall Table 1f, k stands for the quantity of leaders). Meanwhile, nodes in the set of $V_{x}$ will be removed by these leaders after a period of time regardless of their actual node states.

From the discussion above, we can draw 4 problems. First, an approach for estimating the severity of packet loss of link must be found. Second, faulty nodes must be prevented to make any decision on changing the size of cluster. Third, the uniqueness of leader node should be guaranteed. Finally, the leader need an approach to find faulty nodes and do responsible troubleshooting. These problems can be formulated as follows:

(a) Link Estimate Problem (LEP)

*Given:* A local node $v_{i}$ and a remote node $v_{j}$.

*Problem:* Find a network indicator $I_{n}\left(v_{i},v_{j}\right)$ and a function $s_{l}^{\prime }:I_{n}\to S_{n}$ such that the misrecognition rate of link state $\sigma_{l}$ is minimized, which can be expressed as:

$$\sigma_{l}\left(v_{i},v_{j},I_{n}\right):=P\left(s_{l}^{\prime }\left(I_{n}\right)!=s_{l}\left(v_{i},v_{j}\right)\right),\;\min\sigma_{l}.$$

(b) Self Checking Problem (SCP)

*Given:* A local node *v*.

*Problem:* Find a node indicator $I_{v}\left(v\right)$ and a function $s_{n}^{\prime }:I_{v}\to S_{n}$ such that the misrecognition rate of node state $\sigma_{n}$ is minimized, which can be expressed as:

$$\sigma_{n}\left(v,I_{v}\right)=P\left(s_{n}^{\prime }\left(I_{v}\right)!=s_{n}\left(v\right)\right),\;\min\sigma_{n}.$$

(c) Leader Uniqueness Problem (LUP)

*Given:* The node set *V*, the observation of connectivity from node $v_{i}$ to $v_{j}$ denoted by $s_{l}^{\prime }\left(v_{i},v_{j}\right)$.

*Problem:* Find a new implementation of function $r_{c}$ such that at a specified time it is guaranteed that the quantity of leaders in the cluster is no more than 1, which can be expressed as k⩽1.

(d) Faulty Nodes Determination Problem (FNDP)

*Given:* The node set *V*, the observation of connectivity from node $v_{i}$ to $v_{j}$ denoted by $s_{l}^{\prime }$ and the candidate rule $r_{c}$.

*Problem:* Find a new implementation of function $r_{d}$ such that reliability of the removing action is maximized. A reliable removing action can be defined as removing only faulty nodes by the leader.

## 4. The PingBasedDown Algorithm

The PingBasedDown Algorithm is a distributed solution that helps to detect problematic nodes reliably, which therefore enhance the availability of decentralized clusters who use Gossip protocol as their main protocol to manage their membership. Each node in the cluster implements the full function of this algorithm.

In our algorithm, we first collect enough data, which can be potentially used as network indicators $I_{n}$, from application layer. After appropriately preprocessing, we bring them to the first model called Link Evaluation Model which is designed to estimate the link quality with the consideration of stochastic packet loss over TCP protocol. As for other message-based protocols such as UDP, the packet loss rate can be estimated simply using the ping-pong tests without this model so that we do not discuss them in this paper. The link evaluation results are then be used by the next model called Decision Model. It firstly evaluates the healthy status of the local and remote nodes and then determines the leadership. Finally, the node chosen as leader executes the faulty nodes selected by this model.

**Figure 2:**
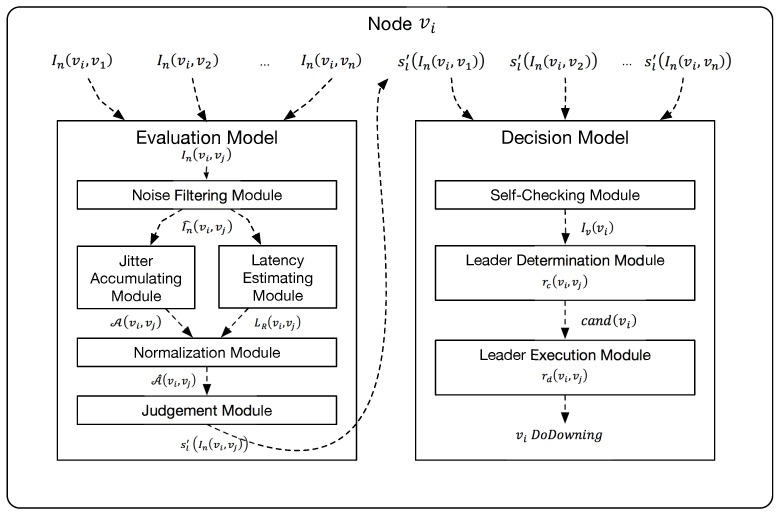
Algorithm overview.

Figure 2 shows the overall solution of the PingBasedDown algorithm.

In preparation for describing in detail, we define some operators on the vector and set:

**Definition** **1.** (FindFirst: $X^{m}\times I\to I$) Giving a vector $X=(x_{1},x_{2},...,x_{m})$, FindFirst(X,α) is defined as returning the subscript of the first value in X that equals to α. For example, suppose X=(1,3,5,5,4), FindFirst(X,5)=2 because $x_{2}$ is the first element in X that equals to 5.

**Definition** **2.** (RemoveAt: $X^{m}\times I\to X^{m-1}$) Giving a vector $X=(x_{0},x_{1},x_{2},...,x_{m})$ and a subscript i,0⩽i⩽m, $RemoveAt\left(X,i\right)=\left(x_{0},x_{1},x_{2},...,x_{i-1},x_{i+1},...,x_{m}\right)$. For example, suppose X=(1,3,5,5,4), RemoveAt(X,3)=(1,3,5,4).

Recall Section 3.3, to solve our problem we should first find a network indicator $I_{n}\left(v_{i},v_{j}\right)$, which is sensitive to packet loss and hence has the ability to estimate the quality of link.

After the analysis of the collected data over TCP protocol with different packet loss rate, we find that the round-trip-time (RTT) of a TCP message is especially sensitive to stochastically packet loss. Here the RTT of a RPC message means time between a certain kind of message and its reply. We then fetch this feature and use it as the network indicator.

To construct the input, i.e., $I_{n}\left(v_{i},v_{j}\right)$, of our model, the node $v_{i}$ keeps collecting the most recent $N_{w}$ groups of timestamps of communication records to $v_{j}$. The timestamp group consists of the sending timestamp of a message and the receiving timestamp of its reply. We then calculate a RTT by subtract the two timestamps. Consider that it may take some time for a remote node $v_{j}$ to process some of the messages, we should subtract the processing durations from corresponded RTTs. We denote $T_{r}\left(v_{i},v_{j}\right)$ to be the vector of collected RTT from $v_{i}$ to $v_{j}$, $T_{p}$ to be a vector of processing delay corresponding to the vector of $T_{r}$. Then, the notations of $T_{r}$, $T_{p}$ and the indicator $I_{n}$ can be expressed as follows.

$$\begin{array}{cc} & T_{r}\left(v_{i},v_{j}\right),T_{p}\left(v_{i},v_{j}\right):V\times V\to R_{+}^{N_{w}},\\ T_{r}\left(v_{i},v_{j}\right) & =\mathrm{The}\;\mathrm{most}\;\mathrm{recently}\;\mathrm{collected}\;N_{w}\;\mathrm{groups}\;\mathrm{of}\;\mathrm{round}-\mathrm{trip}-\mathrm{times},\\ T_{p}\left(v_{i},v_{j}\right) & =\mathrm{The}\;\mathrm{processing}\;\mathrm{time}\;\mathrm{of}\;\mathrm{messages}\;\mathrm{corresponding}\;\mathrm{to}\;T_{r}\left(v_{i},v_{j}\right),\\ I_{n}\left(v_{i},v_{j}\right) & =T_{r}\left(v_{i},v_{j}\right)-T_{p}\left(v_{i},v_{j}\right). \end{array}$$

A simplified approach to construct the input is to collect the RTTs of messages which are supposed to be replied immediately, e.g., heartbeat messages. In this case, the indicator can be $I_{n}\left(v_{i},v_{j}\right)=T_{r}\left(v_{i},v_{j}\right)$.

### 4.1. Link Evaluation Model

Link evaluation model is proposed to evaluate the link quality and solve the LEP. Specifically, this model provides an implementation of function $s_{l}^{\prime }$. In addition to the two original states, a fuzzy state Pending is introduced to avoid any arbitrary judgments. This model consists of the following five modules and Table 3 shows the parameters of this model. First is Noise filtering module, which filters the noise brought by applications from the collected RTTs. The filtered input then goes to the next module named Jitter accumulating module to quantify the jitter. The filtered input also goes to the module named Latency estimating module, which estimates the pure latency, i.e., latency without processing or retransmitting delay, of the link. The quantified jitter and the estimated latency then go through the Normalization module to calculate a normalized result. Finally, the result is compared with two thresholds to evaluate the state of the link. Next, we make detailed description on these modules.

#### 4.1.1. Noise Filtering Module

Since our system works upon application layer, the input of this model $I_{n}\left(v_{i},v_{j}\right)$ is expected to contain noises brought by the application, e.g., garbage collection or thread scheduling process. This module is used to preprocess the input to eliminate the impact of noises. For different scenarios, different implementations of noise filtering modules can be implemented, e.g., removing a part of highest numbers from collected RTTs. Algorithm 1 shows an implementation of noise filtering process. We denote the function of this process as follows,

(4) $$\begin{array}{c} NF:R^{N_{w}}\to R^{\left[N_{w}\times \left(1-F_{S}\right)\right]} \end{array}$$

Then the output $\hat{I_{n}}\left(v_{i},v_{j}\right)=NF\left(I_{n}\left(v_{i},v_{j}\right)\right)$ will be used in next steps.

**Algorithm 1:** Simple noise filtering algorithm.
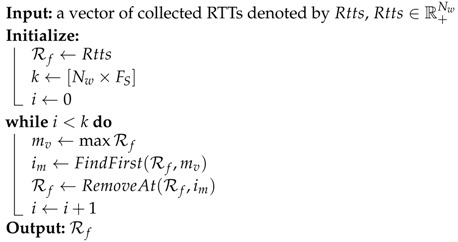


#### 4.1.2. Jitter Accumulating Module

Calculating the variance of data is a commonly used approach to measure the jitter. However, this method is inaccurate in some cases. For example, the two vectors $d_{1}=\left(1,1,1,100,100,100\right)$ and $d_{2}=\left(1,100,1,100,1,100\right)$ have the same variance, but what we want is that the jitter of $d_{2}$ is higher than that of $d_{1}$. Therefore, in this module, we quantify the jitter of $\hat{I_{n}}\left(v_{i},v_{j}\right)$ by accumulating the quantified variation rate. Let A denote the quantification result. We quantify the jitter by the following steps:

Giving a vector of numbers $X\in R^{N_{w}}$, we use first-order difference of this vector ΔX to extract the rate of variation, i.e., jitter. Then we obtain the quantified jitter by accumulating the absolute value of ΔX. The output of this module A can then be represented as follows:

(5) $$A:R^{N_{w}}\to R,\;A\left(X\right)=\sum \left|\Delta X\right|$$

#### 4.1.3. Latency Estimating Module

In order to make the algorithm adapt to different levels of latency, this module is proposed to estimate the pure latency of the link. The pure latency denoted by $L_{R}$ means how long a RTT of the message is without triggering the retransmission mechanism. Through a normal link from $v_{i}$ to $v_{j}$, $L_{R}$ is expected to be: $L_{R}\left(v_{i},v_{j}\right)=E\left(\hat{I_{n}}\left(v_{i},v_{j}\right)\right)$, where $E:R^{N_{w}}\to R$ is the operator of mathematical expectation. However, when messages transmit through an abnormal link with stochastic packet loss, the RTT sometimes may be much longer than the pure latency because of the retransmit mechanism. Thus, we introduce an approach. It cuts off the bigger part of the collected RTTs which are supposed to be caused by retransmission process. Then it calculates the average value of the rest, i.e., the smaller part, of RTTs which are supposed to be transmitted without packet loss. This average value is used as the estimate of pure latency. The cut off action is similar to that of the noise filtering algorithm presented in Algorithm 1 with the replacement from $F_{S}$ to $L_{pos}$.

#### 4.1.4. Normalization Module

From the algorithm of the jitter accumulating module, we can find that the value of the accumulated result has a strong correlation with the length of the vector, denoted by dim(A), and the level of the link latency. This brings difficulty on the judgement of link quality. The Normalization module adjusts this value to a notionally common non-dimensional scale using the formula as follows,

(6) $$\hat{A}=\frac{A}{L_{R}\times \dim\left(A\right)}$$

With the help of this module, no matter what level of link latency is and how long the vector is, the evaluation result for links with same packet loss rates should be approximately within a same range.

#### 4.1.5. Judgement Module

Recall the very first of this section, we define 3 states of link quality:

- Healthy, which stands for normally working link without packet loss;
- Unhealthy, it stands for abnormal link with packet losses;
- Pending, which stands for fuzzy link which may need further detection.

To determine which state the link should be, we compare the $\hat{A}$ with two thresholds, namely safe threshold and alert threshold, denoted by $T_{safe}$ and $T_{alert}$, respectively. In addition, the link status is determined by the following equation:

(7) $$I_{n}\left(v_{i},v_{j}\right)=\left\{\begin{array}{cc} Unhealthy, & \hat{A}⩾T_{alert},\\ Pending, & T_{alert}>\hat{A}>T_{safe},\\ Healthy, & \hat{A}⩽T_{safe}. \end{array}\right.$$

Two parameters of $T_{safe}$ and $T_{alert}$ determine the sensitivity of Link Evaluation Model on link failures. A higher $T_{alert}$ makes the model more stable and decreases the false alarm rate when working on noisy networks. However, an exorbitant $T_{alert}$ also makes the model hard to detect a link failure. Moreover, when the normalized accumulated jitter $\hat{A}$ is lower but very closed to $T_{alert}$, it indicates that the link quality is fuzzy. To make our model more robust, we must prevent giving a Healthy mark on fuzzy links. Therefore, we propose the $T_{safe}$ threshold. A lower $T_{safe}$ makes the model give a Healthy mark of a link more strictly. In the datacenter environment where the nodes in the cluster are physically closed to each other, we believe that the jitter rate of latency there is low and therefore a low $T_{safe}$ shall be set. As for other scenarios such as cloud services, $T_{safe}$ shall be set to a higher value to make the model properly working. These two thresholds can be determined either statically by empirical values or dynamically by adaptive algorithms. In the simulation, we set the thresholds statically based on a long statistical data.

### 4.2. Decision Model

With the link evaluation results, the decision model is proposed to solve the following three problems, first is whether a node itself is healthy(SCP), second is whether a node is the leader who is responsible for removing faulty nodes(LUP), third is which nodes are faulty(FNDP).

**Figure 3:**
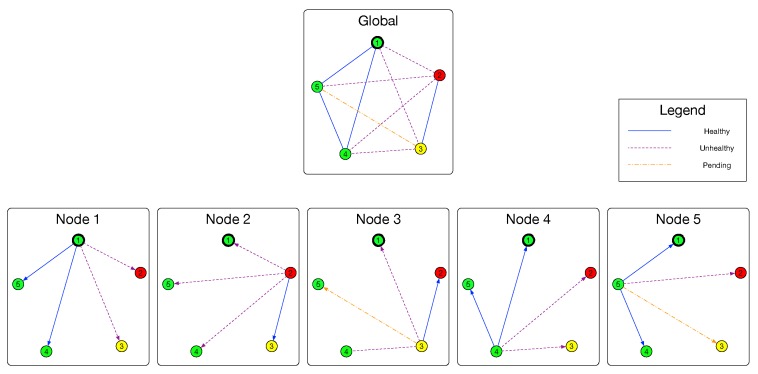
Example of global connectivity graph and sub connectivity graphs. In this example, $E^{\prime }\left(v_{3}\right)=\left\{\left(v_{3},v_{2}\right)\right\}$ and ${\bar{E}}^{\prime }\left(v_{3}\right)=\left\{\left(v_{3},v_{1}\right),\left(v_{3},v_{4}\right)\right\}$

We denote the cluster (nodes and its links) to be a simple undirected graph G=(V,E), where $E=\{\left(v_{i},v_{j}\right):\forall v_{i},v_{j}\in V,s_{l}\left(v_{i},v_{j}\right)=Healthy\}$. As shown in Figure 3, with the help of evaluation model, a node $v_{i}$ can build a group of subgraphs $G^{\prime }\left(v_{i}\right)=\left(V,E^{\prime }\left(v_{i}\right)\right)$ and ${\bar{G}}^{\prime }\left(v_{i}\right)=\left(V,{\bar{E}}^{\prime }\left(v_{i}\right)\right)$ with the result of Evaluation model. Similar to the relationship between $s_{l}$ and $s_{l}^{\prime }$, $E^{\prime }$ (and also ${\bar{E}}^{\prime }$) is the observation of *E*. If an edge is in the set $E^{\prime }$, the evaluation result of this link is Healthy. As for edges existing in ${\bar{E}}^{\prime }$, their evaluation results are Unhealthy. If an edge $\left(v_{i},v_{j}\right)$ is neither in $E^{\prime }\left(v_{i}\right)$ nor in ${\bar{E}}^{\prime }\left(v_{i}\right)$, its evaluation result is Pending. Formally, $E^{\prime }$ and ${\bar{E}}^{\prime }$ are defined as follows:

(8) $$\begin{array}{cc} E^{\prime }\left(v_{i}\right) & :=\{\left(v_{i},v\right):\forall v\in V,s_{l}^{\prime }\left(I_{n}\left(v_{i},v\right)\right)=Healthy\}, \end{array}$$

(9) $$\begin{array}{cc} {\bar{E}}^{\prime }\left(v_{i}\right) & :=\{\left(v_{i},v\right):\forall v\in V,s_{l}^{\prime }\left(I_{n}\left(v_{i},v\right)\right)=Unhealthy\}. \end{array}$$

Next, we present some definitions about the node state.

**Definition** **3.** *Healthy node set. W is the healthy node set if and only if the following 3 conditions are satisfied: (1) $\forall v_{1},v_{2}\in W$, $(v_{1},v_{2})\in E$; (2) $\left|W|⩾[\frac{V}{2}\right]$; (3) ¬∃v∈V s.t. $\forall v_{h}\in W,\left(v,v_{h}\right)\in E$*.

**Definition** **4.** *Healthy node. A node v is Healthy if and only if v∈W*.

**Lemma** **1.** *When a cluster is normally working, if a node $v_{i}$ is healthy, the degree of $v_{i}$ in G must be greater than $[\frac{\left|V\right|}{2}]-1$*.

(10) $$(1),\;s_{n}\left(v_{i}\right)=Healthy\Rightarrow deg\left(v_{i}\right)>\left[\frac{\left|V\right|}{2}\right]-1$$

**Proof.**  Let $V_{h}:=\left\{v:v\in V,s_{n}\left(v\right)=Healthy\right\}$, $V_{h}^{\prime }:=V_{h}\setminus \left\{v_{i}\right\}$. Because of (1), we have $|V_{h}|>\left[\frac{\left|V\right|}{2}\right]$, and according to the definition of $V_{h}^{\prime }$, we have:

(11) $$|V_{h}^{\prime }|>\left[\frac{\left|V\right|}{2}\right]-1$$

With Definition 4, we have $deg\left(v_{i}\right)=\left|V_{h}^{\prime }\right|-1$. Combining with (11), we have $deg\left(v_{i}\right)>\left[\frac{\left|V\right|}{2}\right]-1$. ☐

**Lemma** **2.** *A node $v_{i}$ is unhealthy, if and only if the degree of $v_{i}$ in G must lower than $\left[\frac{\left|V\right|}{2}\right]$.*

(12) $$(1),\;s_{n}\left(v_{i}\right)=Unhealthy\Leftrightarrow deg\left(v_{i}\right)<\left[\frac{\left|V\right|}{2}\right]$$

From Lemma 1 we see that the condition that $deg\left(v_{i}\right)>\left[\frac{\left|V\right|}{2}\right]-1$ is only the necessary but not the sufficient condition of that $v_{i}$ is healthy. However, these nodes with degree greater than half of the cluster size are also potentially healthy, which may need further determination. Thus, we make a new definition with this kind of nodes to be PendingHealthy.

**Definition** **5.** *If the degree of a node v in G is greater than $[\frac{\left|V\right|}{2}]-1$, we say that it is PendingHealthy.*

(13) $$deg\left(v_{i}\right)>\left[\frac{\left|V\right|}{2}\right]-1\Leftrightarrow v_{i}\;\mathrm{is}\;PendingHealthy$$

Particularly, when the transitivity is applicable, if a node is PendingHealty, it must be Healthy.

**Theorem** **1.** *When the transitivity is applicable, if a node is PendingHealthy, it is Healthy. This can be expressed as:*

(14) $$(1),\;(2),\;deg\left(v_{i}\right)>\left[\frac{\left|V\right|}{2}\right]-1\Rightarrow s_{n}\left(v_{i}\right)=Healthy$$

**Proof.**  We let $V_{h}^{\prime }:=\left\{v:v\in V,s_{l}\left(v_{i},v\right)=Healthy\right\}\cup \left\{v_{i}\right\}$. For any two remote nodes $v_{j}$ and $v_{k}$ in $V_{h}^{\prime }$, according to the transitivity (2), the link quality of $v_{j}$ to $v_{k}$ can be inferred by the local node $v_{i}$, which can be expressed as:

(15) $$\forall v_{j},v_{k}\in V_{h}^{\prime }\setminus \left\{v_{i}\right\},\;s_{l}\left(v_{j},v_{k}\right)=Healthy$$

Because the local node $v_{i}$ is PendingHealthy, the degree of $v_{i}$ is greater than $[\frac{\left|V\right|}{2}]-1$ so that the size of $V_{h}^{\prime }$ is greater than half of the cluster’s size: $|V_{h}^{\prime }|>\left[\frac{\left|V\right|}{2}\right]$. (15) means that the nodes in $|V_{h}^{\prime }|$ are fully connected. Combine with Definition 4, the state of $v_{i}$ is Healthy, i.e., $s_{n}\left(v_{i}\right)=Healthy$. ☐

We use the following 3 modules to solve the problem of SCP, LUP and FNDP. They are used to check the state of local node, ensure the uniqueness of leader, construct a global $G^{\prime }$ as an approximation of graph *G* and remove faulty nodes based on $G^{\prime }$, respectively.

#### 4.2.1. Self-Checking Module

At any time, a faulty node should not be selected as the leader. However, the evaluation model or other failure detection mechanisms cannot sense which side, i.e., whether themselves or their peers, is faulty. We thereby design this module to do self-checking and if one’s self-checking procedure indicates that itself is faulty, it will abandon all the next steps and report this issue to the upper applications. More specifically, this module proposes the indicator $I_{v}$ and the function of $s_{n}^{\prime }$.

With the basis of Lemma 1, Lemma 2 and Definition 5, we come up with the idea of this module that, if the majority of the nodes in the cluster announce that $v_{f}\left(v_{f}\in V\right)$ is the faulty one using the evaluation module, $v_{f}$ should be unhealthy. Although it is possible that all the announcers are unhealthy, however, in that case we can say that most of the nodes in the cluster have failed so that the cluster is totally out of function and it would be meaningless to discuss the reliability and availability. On the contrary of the condition of unhealthy, if the majority ones believe that $v_{w}$ is healthy, $v_{w}$ should be healthy although it is actually PendingHealthy which we have already discussed about.

This idea can then be described as:

(16) $$\left\{\begin{array}{cc} I_{v}\left(v_{i}\right) & :=\{v:v\in V\setminus \left\{v_{i}\right\},\;\left(v,v_{i}\right)\in E^{\prime }\left(v\right)\},\\ \bar{I_{v}}\left(v_{i}\right) & :=\{v:v\in V\setminus \left\{v_{i}\right\},\;\left(v,v_{i}\right)\in {\bar{E}}^{\prime }\left(v\right)\},\\ s_{n}^{\prime }\left(I_{v}\right) & =\left\{\begin{array}{cc} Unhealthy, & |\bar{I_{v}}|>\left[\frac{\left|V\right|}{2}\right],\\ Healthy, & |I_{v}|>\left[\frac{\left|V\right|}{2}\right]-1,\\ Pending, & \mathrm{otherwise}. \end{array}\right. \end{array}\right.$$

Unfortunately, the self-checking process is performed in $v_{i}$, who has no knowledge about $E^{\prime }\left(v\right)$ and ${\bar{E}}^{\prime }\left(v\right)$, $\forall v\in V\setminus \{v_{i}\}$. We thereby consider that $s_{l}\left(v,v_{i}\right)=s_{l}\left(v_{i},v\right)$. Because $s_{l}^{\prime }\left(I_{n}\left(v,v_{i}\right)\right)=s_{l}\left(v,v_{i}\right)$ is expected, $s_{l}^{\prime }\left(I_{n}\left(v_{i},v\right)\right)=s_{l}^{\prime }\left(I_{n}\left(v,v_{i}\right)\right)$ is also expected due to transitivity. Combine with (8) and (9), we have the following theorem:

**Theorem** **2.** *If $\left(v_{i},v_{j}\right)\in E^{\prime }\left(v_{i}\right)$ then $\left(v_{j},v_{i}\right)\in E^{\prime }\left(v_{j}\right)$ and if $\left(v_{i},v_{j}\right)\in {\bar{E}}^{\prime }\left(v_{i}\right)$ then $\left(v_{j},v_{i}\right)\in {\bar{E}}^{\prime }\left(v_{j}\right)$*.

With Theorem 2, the function of $s_{n}^{\prime }$ can be easily calculated locally because (16) can be converted to:

(17) $$\begin{array}{c} \left\{\begin{array}{cc} I_{v}\left(v_{i}\right) & =\{v:v\in V\setminus \left\{v_{i}\right\},\left(v_{i},v\right)\in E^{\prime }\left(v_{i}\right)\},\\ \bar{I_{v}}\left(v_{i}\right) & =\{v:v\in V\setminus \left\{v_{i}\right\},\;\left(v_{i},v\right)\in {\bar{E}}^{\prime }\left(v_{i}\right)\}. \end{array}\right. \end{array}$$

If the self-checking result is healthy, this node should go forward to leader determination module. Otherwise, the node may suffer from network failure and it should handle this issue. If the result is pending, the node may do nothing but wait for more reliable link evaluation results.

#### 4.2.2. Leader Determination Module

This module is to make healthy nodes find the cluster leader without election, i.e., find the function $r_{c}$. Recall Table 1 (f), each node is given a unique id by function h(v). With the help of this function, the main idea of this module can be described as choosing the node from the healthy set with the minimal unique id to be the leader. Therefore, we define the basic candidate rule $r_{c}$ to be:

(18) $$r_{c}\left(v_{i},v_{j}\right)=\left\{\begin{array}{cc} TRUE, & iff\;s_{n}^{\prime }\left(I_{v}\left(v_{j}\right)\right)=Healthy,\\ FALSE, & otherwise. \end{array}\right.$$

However, in this case of the basic candidate rule, we can find that one $v_{i}$ must obtain all remote nodes’ healthy states, i.e., $s_{n}^{\prime }\left(I_{v}\left(v_{j}\right)\right)$ for all $v_{j}\in V\setminus \left\{v_{i}\right\}$, which cannot be directly acquired locally. For this reason, an approach is needed to get or infer the status of remote nodes. Here we need to consider about the transitivity. Recall Section 3.2, this property is applicable in most cases but do have exceptions. Hence, we propose two different approaches classified by the applicability of this property.

The first approach is to infer healthy states of remote nodes. In the most common conditions that transitivity is applicable, the two following theorems that can be proved:

**Theorem** **3.** *When transitivity is applicable, for an arbitrary node $v_{t}$, if it has a healthy link that connected to a healthy node $v_{s}$, $v_{t}$ must be healthy. This can be formulated as:*

$$\forall v_{s},v_{t}\in V:\left\{\begin{array}{c} s_{n}\left(v_{s}\right)=Healthy\\ s_{l}\left(v_{s},v_{t}\right)=Healthy \end{array}\right\}\Rightarrow s_{n}\left(v_{t}\right)=Healthy$$

**Proof.**  Because $s_{n}\left(v_{s}\right)=Healthy$, according to Definition 4, we have:

(19) $$\exists V_{h}^{\prime }\;s.t.\left\{\begin{array}{c} |V_{h}^{\prime }|>\left[\frac{\left|V\right|}{2}\right]-1,\\ \mathrm{nodes}\;\mathrm{in}\;V_{h}^{\prime }\;\mathrm{are}\;\mathrm{fully}\;\mathrm{connected},\\ v_{s}\in V_{h}^{\prime }. \end{array}\right.$$

Combine with (19), transitivity (2), and the fact that $s_{l}\left(v_{s},v_{t}\right)=Healthy$, it can be inferred that $\forall v\in V_{h}^{\prime }\setminus \left\{v_{t}\right\},s_{l}\left(v,v_{t}\right)=Healthy$. Thus, we have:

(20) $$deg\left(v_{t}\right)>\left[\frac{\left|V\right|}{2}\right]-1$$

According to Theorem 1 and (20), the state of $v_{t}$ can be demonstrated: $s_{n}\left(v_{t}\right)=Healthy$. ☐

**Lemma** **3.** *(With transitivity) For three nodes $v_{i}$, $v_{j}$ and $v_{k}$, if the link state from $v_{i}$ to $v_{j}$ is Healthy and that from $v_{i}$ to $v_{k}$ is Unhealthy, it can be inferred that the link state from $v_{j}$ to $v_{k}$ is Unhealthy. This lemma can be formulated to:*

$$\exists v_{i},v_{j},v_{k}\in V\;s.t.\;\left\{\begin{array}{c} s_{l}\left(v_{i},v_{j}\right)=Healthy\\ s_{l}\left(v_{i},v_{k}\right)=Unhealthy \end{array}\right\}\Rightarrow s_{l}\left(v_{j},v_{k}\right)=Unhealthy$$

**Theorem** **4.** *(With transitivity) For two nodes $v_{s}$ and $v_{t}$, if the state of $v_{s}$ is Healthy, and the link between $v_{s}$ and $v_{t}$ is Unhealthy, it can be inferred that the state of $v_{t}$ is Unhealthy.*

$$\exists v_{s},v_{t}\in V\;s.t.\;\left\{\begin{array}{c} s_{n}\left(v_{s}\right)=Healthy\\ s_{l}\left(v_{s},v_{t}\right)=Unhealthy \end{array}\right\}\Rightarrow s_{n}\left(v_{t}\right)=Unhealthy$$

Theorem 4 can be proved similar to the proof of Theorem 3.

**Proof.**  According to Lemma 3 and (19), because the link status $s_{l}\left(v_{s},v_{t}\right)=Unhealthy$, we have inferred that $\forall v\in V_{h}^{\prime }:s_{l}\left(v,v_{t}\right)=Unhealthy$, which also can be expressed as $deg\left(v_{t}\right)<\left[\frac{\left|V\right|}{2}\right]$. Combine with Lemma 2, we can conclude that $s_{n}\left(v_{t}\right)=Unhealthy$. ☐

With Theorems 3 and 4, the state of a remote node can be easily inferred from the link evaluation results. In short, when the transitivity is applicable, if the state of a link starts with a healthy node is healthy, the destination remote node is in state of healthy. Hence the candidate rule $r_{c}$ can be redefined to:

(21) $$r_{c}\left(v_{i},v_{j}\right)=\left\{\begin{array}{cc} TRUE, & iff\;s_{l}^{\prime }\left(I_{n}\left(v_{i},v_{j}\right)\right)=Healthy,\\ FALSE, & otherwise. \end{array}\right.$$

The second approach is fetching healthy states from remote nodes. In the condition that the transitivity is not applicable, we have to fetch all the partial topologies from remote nodes and combine them to a global topology. This helps us to choose a unique leader in this module and further help to do execution in the next module.

Recall the very first of this section, partial topology in node $v_{x}$ are denoted by a group of directed graphs $G^{\prime }\left(v_{x}\right)=\left(V,E^{\prime }\left(v_{x}\right)\right)$ and ${\bar{G}}^{\prime }\left(v_{x}\right)=\left(V,{\bar{E}}^{\prime }\left(v_{x}\right)\right)$ and the global topology is denoted by an undirected graph G=(V,E). For an arbitrary healthy node $v_{i}$, the target of this module is to fetch the remote partial $G^{\prime }\left(v\right)$ for $v\in V-\{v_{i}\}$, and combine these $G^{\prime }\left(v\right)$ to an undirected graph $G^{\prime }=\left(V,E^{\prime }\right)$ which is expected to be equal to *G*. When a node successfully constructs $G^{\prime }$, it will be able to calculate the states of nodes using the approach provided in Self-Checking module. The candidate rule can then be defined as:

(22) $$r_{c}\left(v_{i},v_{j}\right)=\left\{\begin{array}{cc} TRUE, & deg_{G^{\prime }}\left(v_{j}\right)>\left[\frac{\left|V\right|}{2}\right]-1,\\ FALSE, & otherwise. \end{array}\right.$$

To introduce our approach in detail, we here make definitions on Healthy observation set and Unhealthy observation set of nodes.

**Definition** **6.** Healthy observation set of an arbitrary node $v_{x}$ is the set of nodes that the link between them and $v_{x}$ is healthy, which can be expressed as $S_{h}\left(v_{x}\right):=\left\{v:v\in V\setminus \left\{v_{x}\right\},s_{l}\left(v_{x},v\right)=Healthy\right\}$.

**Definition** **7.** *Unhealthy observation set is defined as $v_{x}$: $\bar{S_{h}}\left(v_{x}\right):=\left\{v:v\in V\setminus \left\{v_{x}\right\},s_{l}\left(v_{x},v\right)=Unhealthy\right\}$*.

For implementation, we also use $s_{l}^{\prime }\left(I_{n}\left(v_{i},v_{j}\right)\right)$ to approximate $s_{l}\left(v_{i},v_{j}\right)$ in order to obtain the observed $S_{h}$ and $\bar{S_{h}}$ in each node.

To combine the partial topologies into a global topology, we have to deal with the following issues (suppose the local node is $v_{l}$). First, $v_{l}$ fetches partial topologies from the nodes in $S_{h}\left(v_{l}\right)$ directly. Then, $v_{l}$ asks for partial topologies of unreachable nodes, i.e., nodes in $\bar{S_{h}}\left(v_{l}\right)$, with the help of nodes in $S_{h}\left(v_{i}\right)$. While fetching partial topologies, handle the ask timeout. While combining the partial topologies, check for and resolve the conflict state of a certain edge from two sides of nodes. Finally, give the combination result.

Algorithms 2–4 shows the full workflow of this module.

Algorithm 2 shows the workflow of fetching and combining process. This process is started by the initiator who tries to acquire the global topology. It first initializes a pair of mutable graphs $\left(G^{\prime },{\bar{G}}^{\prime }\right)$, which will eventually hold the combination result, i.e., global graph; a mutable set $V_{seen}$, which indicates whose sub connectivity graphs have been combined into the the intermediate $G^{\prime }$ and ${\bar{G}}^{\prime }$, an immutable Healthy observation set $S_{h}$. For all nodes in $S_{h}\cap V_{t}$, it sends a *AskTopo* RPC to these nodes. If a *ReplyTopo* is replied, try to merge the replied graph $G^{\prime }\left(v_{r}\right),{\bar{G}}^{\prime }\left(v_{r}\right)$ with $G^{\prime },{\bar{G}}^{\prime }$. If merging process succeeded, combine the returned seen set $V_{seen}^{\prime }$ with the local seen set $V_{seen}$. If merging process failed, stop the fetching and combining process immediately and report the conflict issue. If no *ReplyTopo* is replied, mark the edge from the local node to this remote node as Unhealthy and also add this remote node into seen set.

Algorithm 4 shows the workflow when a node receives a *AskTopo* RPC. If the sender of this RPC asks for aid to grab sub connectivity graphs from other nodes in $\bar{S_{h}^{r}}$ (because the sender cannot connect to these nodes), the receiver will invoke FetchAndCombine and set the parameter $V_{t}$ to be $S_{h}\cap \bar{S_{h}^{r}}$. If $V_{t}=\emptyset$, the FetchAndCombine will do nothing but return its own sub graph. After FetchAndCombine process is completed, the receiver will pack the combine result into ReplyTopo message and send back to the sender of AskTopo RPC.

**Algorithm 2:** Fetching and combining algorithm. FetchAndCombine($V,v_{l},G^{\prime },{\bar{G}}^{\prime },V_{t},V_{aid}$).
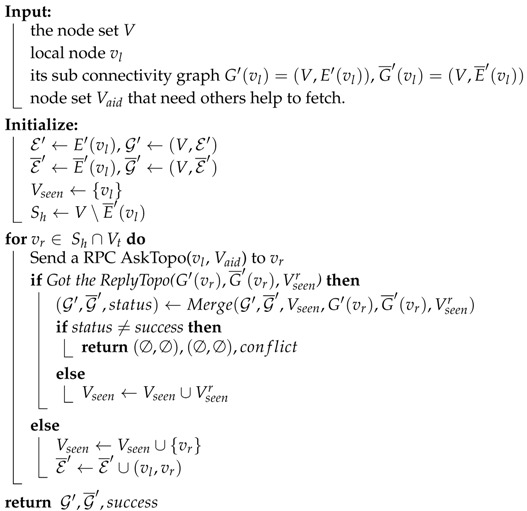


**Algorithm 3:** Merging algorithm. Merge($G_{1},{\bar{G}}_{1},V_{1}^{seen},G_{2},{\bar{G}}_{2},V_{2}^{seen}$).
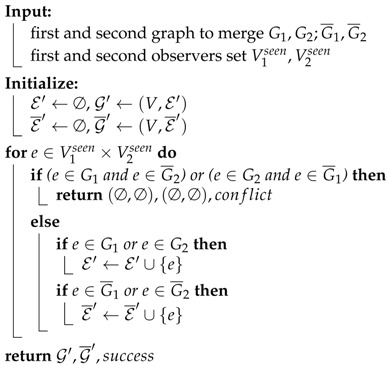


**Algorithm 4:** Replying algorithm.
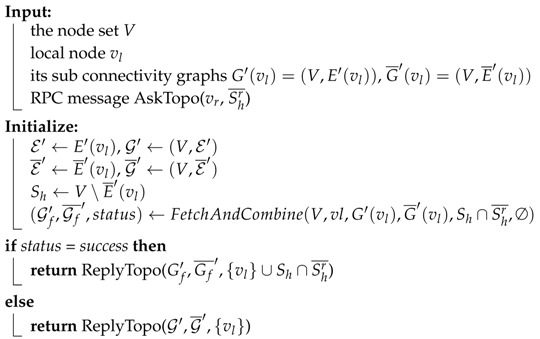


Generally speaking, the workflow is that, the initiator $v_{i}$ starts the fetching process by calling FetchAndCombine($V,v_{i},G^{\prime }\left(v_{i}\right),{\bar{G}}^{\prime }\left(v_{i}\right),S_{h}\left(v_{i}\right),\bar{S_{h}}\left(v_{i}\right)$), which will send AskTopo RPCs to all reachable remote nodes. This will trigger the process of Algorithm 4 in all these remote nodes. All the receivers then try to reply to $v_{i}$ ReplyTopo messages containing their sub connectivity graphs and possibility containing the sub connectivity graphs of nodes cannot be reached by the sender. The function of FetchAndCombine will call the function Merge, which is presented in Algorithm 3, to merge different sub graphs after checking and resolving the conflict. Our default conflict resolving policy is that: if two sides give the detection result of an edge as a Pending and a non-Pending, the resolving result will be the non-Pending result; if two sides give the detection result of an edge as two different non-Pending results, the merging process will stop and give out the result of “conflict”.

For example, if $v_{i}$ and $v_{j}$ detects the edge $\left(v_{i},v_{j}\right)$ to be Healthy and Pending, respectively, after merging, the edge will be marked as Healthy which is to say the edge will appeared in the Healthy edge set $E^{\prime }$; if $v_{i}$ and $v_{j}$ detects the edge $\left(v_{i},v_{j}\right)$ to be Healthy and Unhealthy, respectively, merging will be interrupted. Table 4 shows the detail of this policy.

#### 4.2.3. Leader Execution Module

Leader node chosen by the former module uses leader execution module to find the set of $V_{R}$ and removes these nodes in $V_{R}$ from the cluster. Then, for a leader node $v_{l}$ and an arbitrary remote node $v_{x}$, the downing rule can be described as follows:

(23) $$r_{d}\left(v_{l},v_{x}\right)=\left\{\begin{array}{cc} TRUE, & \mathrm{iff}\;v_{x}\in V_{R},\\ FALSE, & \mathrm{otherwise}. \end{array}\right.$$

We construct $V_{R}$ based on the idea that after the execution, the rest of the nodes can normally transmit data with each other, which is to say they form a complete graph from the perspective of graph theory. So we abstract the objective of this module to be (1) find a maximal clique, with best effort, a maximum clique, of $G^{\prime }$, which means finding a subgraph $K_{p}^{\prime }=\left(V_{m},\left(\begin{array}{c} V_{m}\\ 2 \end{array}\right)\right)$ of $G^{\prime }$ with maximal or maximum nodes satisfying the condition that $K_{p}^{\prime }$ is a complete graph; (2) let $V_{R}:=V\setminus V_{m}$.

The MCP (max clique problem) is a NP-Complete problem [24]. Therefore, we need an algorithm to reduce the computing complexity to make our algorithm available in big clusters. In this section, according to the applicability of transitivity, we propose two algorithms for each case. In the first case with the property, our algorithm will always find a max clique with the computing complexity of O(|V|). While in the second case without the property, we propose simple heuristic algorithm that will find a maximal (maybe max) clique with the computing complexity of $O(|V{|}^{2})$.

The first scenario is that the transitivity is applicable. In this case, the leader $v_{l}$ can find a max clique by a very simple policy of:

(24) $$\begin{array}{c} \mathrm{let}\;V_{m}\leftarrow \left\{v:v\in V\setminus \left\{v_{l}\right\},\left(v_{l},v\right)\in E\right\}\cup \left\{v_{l}\right\},\\ K_{p}=\left(V_{m},\left(\begin{array}{c} V_{m}\\ 2 \end{array}\right)\right),\mathrm{where}\;p=\left|V_{m}\right|. \end{array}$$

This is because the theorem below can be proved.

**Theorem** **5.** With the transitivity, a healthy node and all its peers end with healthy links construct the unique max clique of the global graph G.

(25) $$\begin{array}{c} G=(V,E)\;\mathrm{is}\;\mathrm{an}\;\mathrm{undirected}\;\mathrm{graph}.\\ S_{m}\;\mathrm{is}\;a\;\mathrm{set}\;\mathrm{of}\;\max\;\mathrm{cliques}\;\mathrm{of}\;G.\\ \begin{array}{c} \left.\begin{array}{c} \left(2\right)\\ \exists v\;s.t.\;deg_{G}\left(v\right)>\left[\frac{\left|V\right|}{2}\right]-1 \end{array}\right\}\Rightarrow \left\{\begin{array}{cc} \left(1\right) & |S_{m}|\equiv 1,\\ \left(2\right) & \begin{array}{c} \exists K_{p}=\left(V_{m},\left(\begin{array}{c} V_{m}\\ 2 \end{array}\right)\right),\\ V_{m}=\left\{v_{x}:\left(v,v_{x}\right)\in E\right\}\cup \left\{v\right\}\;s.t.\;K_{p}\in S_{m}. \end{array} \end{array}\right. \end{array} \end{array}$$

**Proof.**  We let $E_{m}=\left(\begin{array}{c} V_{m}\\ 2 \end{array}\right)$, $V_{R}=V\setminus V_{m}$. We also introduce the notations of $K_{p}$, $S_{m}$, $V_{m}$, $E_{m}$, *G*, *V*, *E* in Theorem 5 (25).

Maximal proof:

Because the definition of $V_{R}$ means a set of nodes with unhealthy link state with at least one node in set $V_{m}$, which can be expressed as: $\forall v\in V_{R}:\exists v_{h}\in V_{m}\;s.t.\;\left(v_{h},v\right)\notin E_{m}$. We can then conclude that $\forall v_{f}\in V_{R}:$, the graph $\tilde{G}=\left(V_{m}\cup \left\{v\right\},E_{m}\cup \left\{\left(v_{f},v_{h}\right):v_{h}\in V_{m},\left(v_{f},v_{h}\right)\in E\right\}\right)$ is not a complete graph, which means the graph $K_{p}=\left(V_{m},E_{m}\right)$ is a maximal clique of the graph G.

Maximum and unique proof:

We assume that,

(26) $$\exists K_{x}=\left(V_{x},\left(\begin{array}{c} V_{x}\\ 2 \end{array}\right)\right),K_{x}\neq K_{p},V_{x}\subset V\;s.t.\;x⩾p,\;\mathrm{where}\;x=\left|V_{x}\right|.$$

We denote $V_{p}=V_{R}\cap V_{x}$, $V_{q}=V_{m}\cap V_{x}$. If (26) is true, and because $p>[\frac{\left|V\right|}{2}]-1$, we can infer that $V_{p}\neq \emptyset$, $V_{q}\neq \emptyset$. Then $\exists v_{p},v_{q}$, $v_{p}\in V_{R}$, $v_{q}\in V_{m}$ s.t. $v_{p}\in K_{x}$, $v_{q}\in K_{p}$. Thus we have,

(27) $$\exists v_{p},v_{q},v_{p}\in V_{R},v_{q}\in V_{m}\;s.t.\;\left(v_{p},v_{q}\right)\in E.$$

On the other hand, $\forall v_{p}\in V_{R}$, $\exists v_{q}\in V_{m}$ s.t. $(v_{p},v_{q})\notin E$. According to Lemma 3, we have the inference that,

(28) $$\forall v_{p}\in V_{R},\forall v_{q}\in V_{m},\neg \exists \left(v_{p},v_{q}\right)\;s.t.\;\left(v_{p},v_{q}\right)\in E.$$

(27) conflicts with (28), thereby the assumption (26) is false. Thus, $\neg \exists K_{x}\neq K_{p}$ s.t. x⩾p. ☐

Therefore, in this case, the leader node $v_{l}$ can simply construct the global $G^{\prime }$ from $G^{\prime }\left(v_{l}\right)$ and ${\bar{G}}^{\prime }\left(v_{l}\right)$ to approximate *G*. With a loose policy that a leader will not remove a node with the link state of Pending between them, the $G^{\prime }$ can be built with:

(29) $$G^{\prime }=\left(V,E\right),E^{\prime }=\left(\begin{array}{c} V\\ 2 \end{array}\right)\setminus {\bar{E}}^{\prime }\left(v_{l}\right).$$

Finally, with (24) and (29), we find the set $V_{R}$:

(30) $$V_{R}=\left\{v:\left(v_{l},v\right)\notin E^{\prime }\right\}$$

The second scenario is that the transitivity is not applicable. In this case, we propose an easy-understanding, easy-implementing algorithm that will always find a maximal clique $K_{p}$ of *G*. Our idea is that, from the Self-Checking module, we can see that the healthy state of a node $v_{x}$ has strong correlation with $deg(v_{x})$. We therefore iteratively remove the node $v_{f}$ with worst state, i.e., minimal $deg(v_{f})$ until the rest of the nodes are fully connected. Algorithm 5 shows the process of this algorithm in details.

**Algorithm 5:** Finding a maximal clique.
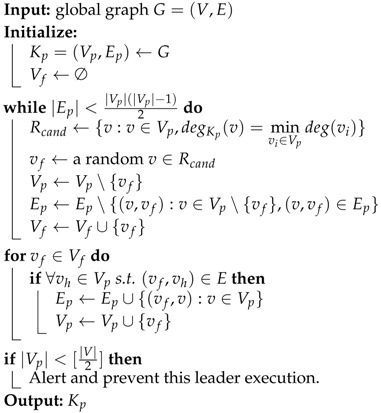


After the leader get the clique $K_{p}=\left(V_{m},\left(\begin{array}{c} V_{m}\\ 2 \end{array}\right)\right)$, it will find the set $V_{R}$ to be:

(31) $$V_{R}=\left\{v:v\notin V_{m}\right\}$$

## 5. Performance Evaluation

### 5.1. Simulation Setup

#### 5.1.1. Baseline Methods

We compare our evaluation model with four algorithms as follows: (1) Φ accrual failure detector (PFD) [8] is a commonly used adaptive failure detector, which assumes that the interval of heartbeat responses follows normal distribution. Specifically, they define a metric of link state ϕ by $\phi =-\log_{10}\left(1-F\left(timeSinceLastHeartbeat\right)\right)$, where *F* is the cumulative distribution function of a normal distribution with mean and standard deviation estimated from historical heartbeat inter-arrival times. By comparing ϕ with a threshold $T_{\phi }$, it gives out the state of a link. (2) Exponential Distribution Failure Detector (EDFD) is an adaptive failure detector [9], which assumes the interval of heartbeat responses follows exponential distribution. Specifically, it defines a metric of link state $E_{d}$ by $E_{d}=F\left(timeSinceLastHeartbeat\right)$, where $F\left(t\right)=1-e^{-\frac{1}{\mu }t}$. By comparing $E_{d}$ with a threshold $T_{ed}$, it gives out the state of a link. (3) 2WFD is an adaptive failure detector [10] that optimizes the Chen FD [7]. It uses two windows with different sizes, i.e., size of $n_{1}$ and $n_{2}$, to store the interval of recent heartbeats. By comparing current time $T_{now}$ and the predicted time $\tau_{l+1}=\max\left(EA_{l+1}^{n_{1}},EA_{l+1}^{n_{2}}\right)+\alpha$, it gives out the state of a link. In the formula above, $EA_{l+1}^{n_{1}}$ and $EA_{l+1}^{n_{1}}$ are the next heartbeat exptected time calculated from the two windows respectively. (4) Calculating the coefficient of variation of network latency is an approach to evaluate the severity of packet loss rate of a link. It quantifies the jitter *C* by calculating the coefficient of variation of the collected RTTs. The coefficient of variation is calculated by: $C=\frac{\;\sigma \;}{\mu }$, where σ stands for the standard deviation, and μ stands for the mean value. By comparing *C* with a threshold $T_{c}$, it gives out the state of a link. We denote this algorithm as CV. Also, we denote our proposed algorithm as AV.

We also compare our system-level testing results with a simulated controller cluster. In the simulated controller cluster, we implement a cluster of nodes which uses the original Downing mechanism, namely AutoDown, which we have already discussed before (Recall Table 2).

#### 5.1.2. Evaluation Metrics

In order to measure the correctness of the link evaluation methods compared with the true link state, we use a well-established and widely-used metric in binary classification to quantify the detection accuracy, i.e., F1-score [25]. Specifically, F1-score is defined based on precision rate and recall rate [25]. Precision rate can be expressed as $P\left(M_{h},N_{h}\right)=\frac{M_{h}}{N_{h}}$. We denote $M_{h}$ to be the number of healthy markers on a healthy link while $N_{h}$ is the number of evaluations on this link. Then, recall rate can be expressed as $R\left(M_{f},N_{f}\right)=\frac{M_{f}}{N_{f}}$. We denote $M_{f}$ to be the number of unhealthy markers on a unhealthy link while $N_{f}$ is the number of evaluations on this faulty link. Based on them, F1 score is defined as: $F1=\frac{2\times P\times R}{P+R}$. A higher F1 score indicates a better performance on an evaluating approach.

To measure the performance of system-level testing results, we focus on the detection rate and mis-kicking rate. The detection rate $R_{D}$ indicates the speed to detect and remove faulty nodes in the cluster. Because in our testing environment, once a faulty node is kicked, it will restart immediately and it takes about $T_{setup}=$ 2 min to startup. We then define the detection rate to be $R_{D}=\frac{N_{fr}\times T_{setup}}{S_{f}\times T_{run}}$, where $S_{f}$ denotes how many faulty nodes are there in this test case, $N_{fr}$ denotes how many times faulty nodes being removed and $T_{run}$ denotes the total testing duration of this test. The mis-kicking rate ${\bar{R}}_{k}$ indicates the severity of incorrect Downing process which causes healthy nodes being removed from the cluster. We define the mis-kicking rate to be $\bar{R_{k}}=\frac{N_{r}-N_{fr}}{N_{r}}$, where $N_{r}$ denotes the total quantity of downing actions. A higher $R_{D}$ and a lower $\bar{R_{k}}$ indicate a better performance.

#### 5.1.3. Simulation Scenarios

We run three groups of experiments to check the accuracy of our algorithm compared with baseline methods. In the first group of experiment, we compare the precision-recall and F1 score with fixed optimized parameter and varying packet loss rate. In the second group of experiment, we compare the precision-recall and F1 score with a fixed packet loss rate and varying parameters, i.e., number of records of RTT and thresholds. In the last group, we apply our algorithm on a real-world product and compare the result with the baseline method.

### 5.2. Evaluation Results

#### 5.2.1. Adaptability of Environment

**Figure 4:**
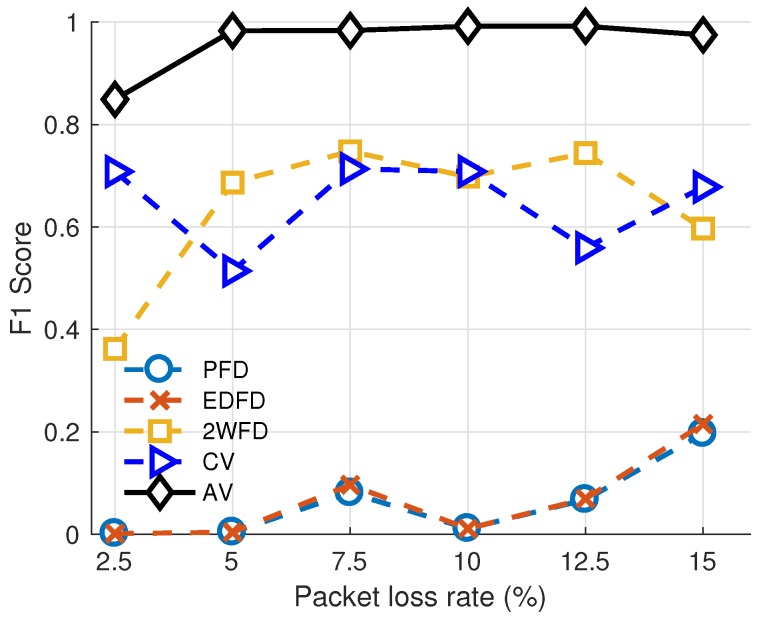
Performance of Evaluation model vs. packet loss rates.

We present the link evaluation results with varying packet loss rate of the three algorithms in Figure 4. This group of experiment indicates the adaptability of each algorithm with different packet loss rates. We fix the parameters of each algorithm. The parameters of each algorithm are set as follows. For PFD, the size of historical heartbeats is set to 100, and the threshold $T_{\phi }$ is set to 0.45. For EDFD, the size of historical heartbeats is set to 1000, and the threshold $T_{ed}$ is set to 0.65. For 2WFD, the size of large window $n_{1}$ is set to 2000, the size of small window $n_{2}$ is set to 10, and the decision time α is set to 0.

For AV and CV, $N_{w}$ (number of records) is set to 30. The thresholds for AV are: $T_{safe}=0.6$, $T_{alert}=1.5$. The threshold for CV is $T_{c}=1.0$. From the results, we can observe that our algorithm achieves the highest F1 score in each network environment, which proves that our evaluation method is accurate in detecting the severity of different packet loss rates.

#### 5.2.2. Impact of Parameters

**Figure 5:**
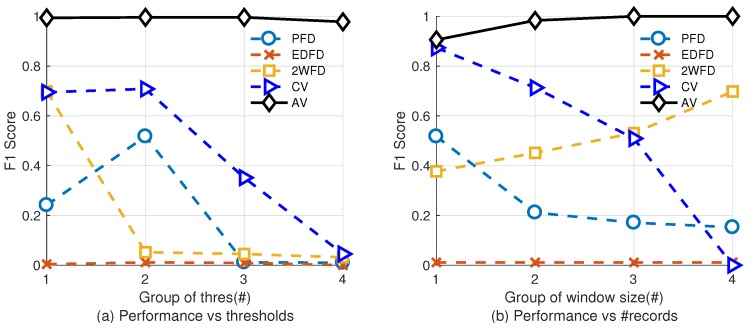
Performance of Evaluation model vs. parameters.

Figure 5 shows the impact of parameters on the performance of each algorithm. In this group of experiment, we fix the packet loss rate to 12.5%. Figure 5a shows the result with varying thresholds. We select 4 groups of typical thresholds for each algorithm. Table 5 shows the thresholds we use. We fix the size of historical heartbeats of PFD, EDFD, 2WFD the same as the latter group of experiment, and fix the length of records $N_{w}$ of AV and CV to be 30. Figure 5b shows the result with varying record sizes. We check four different groups of sizes of records and fix the other parameters as same as the former experiment (Adaptability of Environment).

Table 6 shows the count of records we use in this group of experiment.

Results show that, with different parameters, the performance of our algorithm is higher than the other four algorithms. Specifically, the accuracy can be improved by adjusting the number of records $N_{w}$ to higher values.

Results of Real-world Testing To test and verify the reliability of our algorithm, we apply the algorithm on a product named AgileController. This product is provided by Huawei Inc. It is developed based on a famous open source SDN controller, i.e., OpenDaylight. OpenDaylight supports the feature of constructing a SDN controller cluster to provide most of the advantages brought by distributed systems. Specifically, the OpenDaylight project adopts Akka cluster service, which provides a decentralized and gossip protocol-based membership management service. Thus, we implement PingBasedDown algorithm as a plugin of Akka, and hook this plugin into this product. The AgileController has the ability to automatically restart if it is shut down. The startup process takes about 2 min if restarted. We first construct a cluster of AgileControllers containing several nodes in their simulated production environment. Then we injure failures into some of these nodes with various packet loss rate by the tool named TC of linux operating system. During testing, if one node is removed by a leader, a log containing the information of this removing action, e.g., who is being removed, is generated. After a relatively long period of time, we collect the logging data and calculate $R_{D}$ and $\bar{R_{k}}$ of each test.

The scenario of our simulated production environment is that, each controller runs in a virtual machine. The hosts of these virtual machines are within the same data center with network bandwidth of 1 Gbps. The average transmission delay is around 400 μs. The physical jitter of the network latency is low but the garbage collection process in JVM causes extra jitter when measuring the latency. Based on this environment, we set the parameters of PingBasedDown algorithm as follows. The length of records $N_{w}$ is set to be 30, filter strength $F_{S}$ is set to be 0.15, latency positioning factor $L_{pos}$ is set to be 0.2, and the two thresholds $T_{alert}$, $T_{safe}$ are set to be 17.0 and 2.0, respectively.

During 8 days, we do 15 groups of testing that cover different numbers of nodes, different packet loss rates, different numbers of faulty nodes. To show the benefit of our proposed algorithm, we also provide 6 groups of results with AutoDown algorithm. Table 7 shows the results of this experiment using PingBasedDown algorithm, while Table 8 shows the results using the original AutoDown mechanism provided by Akka. For example, the 7th row in Table 7 means that, we run this group of test with a cluster with 5 nodes. The 1st node and the 3rd node (ordered by h(v) ascendingly) are injured with packet loss failures, and the packet loss rate is set to 15% and 40%, respectively. The result of this group is that, the detection rate $R_{D}$ reaches 61.3% and the mis-kicking rate $\bar{R_{k}}$ is 0.0%. Compared with the baseline method, we can see that when the packet loss rate is as low as 15%, the $R_{D}$ of AutoDown is less than 9.1%, while that of our algorithm ranges from 44.7% to 54.4%. This indicates that our algorithm can detect nodes with packet loss issues faster than AutoDown. The $\bar{R_{k}}$ of AutoDown ranges from 8.9% to 48.1%, which indicates a poor performance on the accuracy of downing action. With our algorithm, the $\bar{R_{k}}$ in all test cases reach a perfect low rate 0.0%, which means that each removing action removes only faulty nodes. Therefore, we can conclude that the results prove the effectiveness and robustness of our proposed algorithm.

## 6. Conclusions

In this work, we propose an algorithm that consists of Evaluation model and Decision model. This algorithm solves the problem of reduced availability in decentralized clusters when nodes occur to randomly drop packets. Driven by the application layer data, the Evaluation model estimates the link and gives relatively accurate evaluation on link quality. With the link evaluations, the Decision model further identifies the only leader. By modeling the cluster to a simple undirected connectivity graph, the leader finds a max clique of this graph. Then, the leader removes the nodes which are not in this clique in which way to make the cluster more stable and available. Classified by the applicability of transitivity, we simplified the NP-Complete maximum clique problem to a linear and a square complexity algorithm. We then evaluate our algorithm with statistical data. Moreover, we implement our solution and adopt it in a real-world product. All these results show that our approach is highly adaptable and available.

## Figures and Tables

**Table 1:** Formal system model.

(a) A set *V* of n nodes (n=|V|) (b) A set $S_{n}$.Two functions $s_{l}:V\times V\to S_{n}$, $s_{n}:V\times V\to S_{n}$. $s_{l}$ maps link to link state; $s_{n}$ maps node to node state. $$\begin{array}{cc} S_{n} & =\{Healthy,Unhealthy\}\\ s_{l}\left(v_{i},v_{j}\right) & =\left\{\begin{array}{cc} Unhealthy, & \mathrm{iff}\;\mathrm{the}\;\mathrm{link}\;\mathrm{from}\;v_{i}\;\mathrm{to}\;v_{j}\;\mathrm{is}\;\mathrm{unhealthy}\\ Healthy, & \mathrm{otherwise} \end{array}\right.\\ s_{n}\left(v\right) & =\left\{\begin{array}{cc} Unhealthy, & \mathrm{iff}\;\mathrm{the}\;\mathrm{node}\;v\;\mathrm{is}\;\mathrm{unhealthy}\\ Healthy, & \mathrm{otherwise} \end{array}\right. \end{array}$$ An equation: (3) $$s_{l}\left(v_{i},v_{j}\right)=s_{l}\left(v_{j},v_{i}\right)$$ (c) A set $S_{R}$ of two reachability states and a function $fs:V\times V\to S_{R}$ that returns the result of failure detecting. $$\begin{array}{cc} S_{R} & =\{Reachable,Unreachable\}\\ fs(v_{i},v_{j}) & =\mathrm{reachability}\;\mathrm{state}\;\mathrm{of}\;v_{j}\;\mathrm{seen}\;\mathrm{by}\;v_{i} \end{array}$$ (d) A procedure GossipTo: $$v_{i}\;GossipTo\;v_{j}:\;\forall v\in V\;\mathrm{let}\;fs\left(v_{j},v\right)\leftarrow fs\left(v_{i},v\right)\;\mathrm{iff}\;fs\left(v_{i},v\right)\;\mathrm{is}\;\mathrm{newer}$$ (e) A function $r_{c}:V\times V\to \left\{TRUE,FALSE\right\}$ used as candidate rule in leader selection process. $$\begin{array}{c} r_{c}\left(v_{i},v_{j}\right)=\left\{\begin{array}{cc} TRUE, & \mathrm{iff}\;v_{j}\;\mathrm{can}\;\mathrm{be}\;a\;\mathrm{candidate}\;\mathrm{from}\;\mathrm{the}\;\mathrm{perspective}\;\mathrm{of}\;v_{i}\\ FALSE, & \mathrm{otherwise} \end{array}\right. \end{array}$$ and a relation $cand:V\to P\left(V\right)\;\mathrm{where}\;v_{k}\in cand\left(v_{i}\right)\;\mathrm{iff}\;r_{c}\left(v_{i},v_{k}\right)=TRUE$ (f) Two functions about non-electoral leader selection h:V↣I, l:V→V $$\begin{array}{cc} h(v_{x}) & =\mathrm{unique}\;\mathrm{identity}\;\mathrm{of}\;v_{x}\\ l(v_{i}) & =\underset{v_{x}\in cand\left(v_{i}\right)}{argmin}h\left(v_{x}\right) \end{array}$$ and an integer k,0<k⩽n, stands for number of leaders at a time: k=|{v:v∈V,l(v)=v}| (g) A function $r_{d}:V\times V\to \left\{TRUE,FALSE\right\}$ used as rule of Downing. A procedure DoDowning $$\begin{array}{cc} r_{d}\left(v_{i},v_{j}\right)= & \left\{\begin{array}{cc} TRUE, & \mathrm{iff}\;l\left(v_{i}\right)=v_{i}\;\mathrm{and}\;v_{i}\;\mathrm{believes}\;v_{j}\;\mathrm{is}\;\mathrm{no}\;\mathrm{longer}\;\mathrm{vaild}\\ FALSE, & \mathrm{otherwise} \end{array}\right.\\ v_{i}\;DoDowning: & \mathrm{let}\;V\leftarrow V\setminus \{v:v\in V,r_{d}\left(v_{i},v\right)=TRUE\} \end{array}$$

**Table 2:** Original implementation of $r_{c}$ and $r_{d}$.

(a) The original implementation of function $r_{c}$: $\begin{array}{c} r_{c}\left(v_{i},v_{j}\right)=\left\{\begin{array}{cc} TRUE, & \mathrm{iff}\;fs(v_{i},v_{j})=Reachable\\ FALSE, & \mathrm{iff}\;fs(v_{i},v_{j})=Unreachable \end{array}\right. \end{array}$ (b) The original implementation of function $r_{d}$: $\begin{array}{c} r_{d}\left(v_{i},v_{j}\right)=\left\{\begin{array}{cc} TRUE, & \mathrm{iff}\;fs(v_{i},v_{j})=Unreachable\;\mathrm{for}\;a\;\mathrm{period}\;\mathrm{of}\;\mathrm{time}\;\mathrm{and}\;\mathrm{do}\;\mathrm{not}\;\mathrm{recover}\\ FALSE, & \mathrm{otherwise}\; \end{array}\right. \end{array}$

**Table 3:** Parameters of Link Evaluation Model.

Notation	Parameter Name	Description
$N_{w}$	History size	How many groups of rtt history we use as the basis.
$F_{S}$	Filter strength	Indicates the strength of filtering, e.g., the proportion of noise in the records.
$L_{pos}$	Latency positioning factor	Indicates rate of pure latency participate in calculating among history of RTTs.
$T_{alert}$	Alert threshold	If the normalized accumulated value is higher than this threshold, mark the link as Unhealthy
$T_{safe}$	Safe threshold	If the normalized accumulated value is lower than this threshold, mark the link as Healthy

**Table 4:** Edge merging policy.

Edge State 1	Edge State 2	Merge Result
Healthy	Healthy	Healthy
Unhealthy	Unhealthy	Unhealthy
Healthy	Pending	Healthy
Unhealthy	Pending	Unhealthy
Pending	Pending	Pending
Healthy	Unhealthy	Conflict

**Table 5:** Thresholds used by experiment on parameters.

Algorithm	Threshold #1	Threshold #2	Threshold #3	Threshold #4
PFD	ϕ=0.05	ϕ=0.25	ϕ=0.45	ϕ=1.5
EDFD	$T_{ed}=0.6$	$T_{ed}=0.7$	$T_{ed}=0.8$	$T_{ed}=0.9$
2WFD	α=0	α=10 ms	α=20 ms	α=30 ms
CV	$T_{c}=0.6$	$T_{c}=1.0$	$T_{c}=1.5$	$T_{c}=2.0$
AV	$T_{safe}=0.6$ $T_{alert}=1.5$	$T_{safe}=1.0$ $T_{alert}=3.0$	$T_{safe}=1.5$ $T_{alert}=6.0$	$T_{safe}=2.0$ $T_{alert}=10.0$

**Table 6:** Window sizes used by experiment on parameters.

Algorithm	Record Size #1	Record Size #2	Record Size #3	Record Size #4
PFD	100	500	1000	2000
EDFD	100	500	1000	2000
2WFD	$n_{1}=100$ $n_{2}=1$	$n_{1}=1000$ $n_{2}=1$	$n_{1}=1000$ $n_{2}=10$	$n_{1}=2000$ $n_{2}=10$
CV	10	30	60	400
AV	10	30	60	400

**Table 7:** The system-level testing results. Each group is tested for 12 h.

Size of Cluster	Faulty Nodes	Packet Loss Rate (%)	Detection Rate $R_{D}\;\left(\%\right)$	Mis-Kicking Rate $\bar{R_{k}}\;\left(\%\right)$
5	1	15	44.7	0
5	1	25	58.3	0
5	1	40	61.9	0
5	1, 3	15, 15	47.5	0
5	1, 3	25, 25	58.6	0
5	1, 3	40, 40	60.2	0
5	1, 3	15, 40	61.3	0
5	1, 3	25, 40	60.8	0
5	1, 3	15, 25	57.5	0
7	1, 2, 3	15, 15, 15	54.4	0
7	1, 2, 3	25, 25, 25	56.1	0
7	1, 2, 3	40, 40, 40	66.4	0
7	1, 2, 3	15, 40, 25	63.1	0
7	1, 2, 3	40, 40, 15	67.2	0
7	1, 2, 3	25, 25, 40	58.3	0

**Table 8:** The system-level testing results with the original AutoDown mechanism of Akka. Each group is tested for 6 h .

Size of Cluster	Faulty Nodes	Packet Loss Rate (%)	Detection Rate $R_{D}\;\left(\%\right)$	Mis-Kicking Rate $\bar{R_{k}}\;\left(\%\right)$
5	3, 4	15, 15	5.0	29.4
5	3, 4	25, 25	32.3	43.4
5	3, 4	40, 40	31.9	8.9
7	4, 5, 6	15, 15, 15	9.1	27.8
7	4, 5, 6	25, 25, 25	50.2	48.1
7	4, 5, 6	40, 40, 40	57.7	33.0
